# Supplementary material for: A library-based approach allows systematic and rapid evaluation of seed region length and reveals design rules for synthetic bacterial small RNAs
Source: iScience. 2024 Aug 20;27(9):110774. doi: 10.1016/j.isci.2024.110774 (PMC11402225; doi:10.1016/j.isci.2024.110774)
Supplement: Document S1. Figures S1–S8 and Tables S1–S4 [file mmc1.pdf]

iScience, Volume *27*

Supplemental information

A library-based approach allows systematic and rapid evaluation of seed region length and reveals design rules for synthetic bacterial small RNAs

Michel Brück, Tania S. Köbel, Sophie Dittmar, Adán A. Ramírez Rojas, Jens Georg, Bork A. Berghoff, and Daniel Schindler

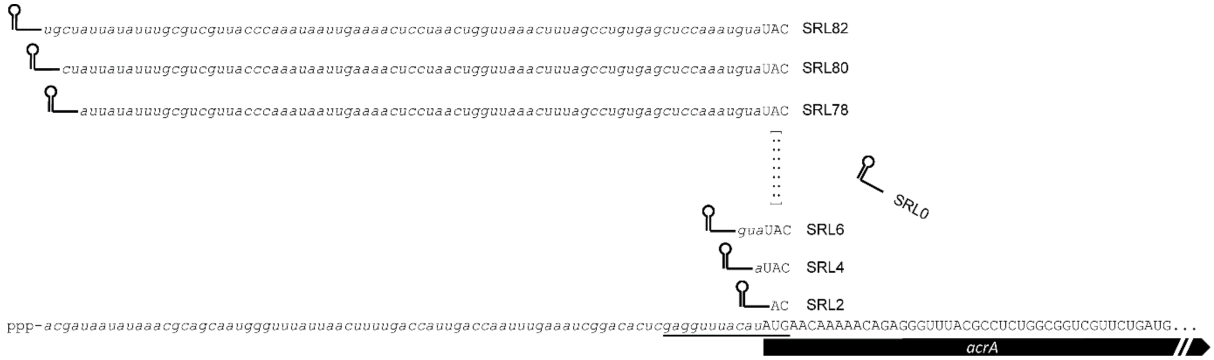

Figure S1 | RybB seed region length library for targeting of *acrA* mRNA, related to Figure 1. The *acrA* 5’ UTR has a length of 79 nt, indicated by lowercase letters. The *acrA* open reading frame is indicated by the black arrow and capital letters. The RybB seed region length (SRL) library includes the start codon and extends to the 5’ end of the transcript in 2-nt increments. The library consists of 42 different lengths, SRL0 represents a RybB scaffold that has no seed region and cannot bind to the *acrA* mRNA. The smaller sRNAs presumably do not bind, but are indicated at their cognate position to visualize the concept of the SRL library. For convenience, only SRL0 to SRL6 and SRL78 to SRL82 are visualized (dots indicate increasing seed region lengths). The translation initiation region (TIR) is underlined in the *acrA* mRNA. The *acrA* mRNA is truncated for visualization.

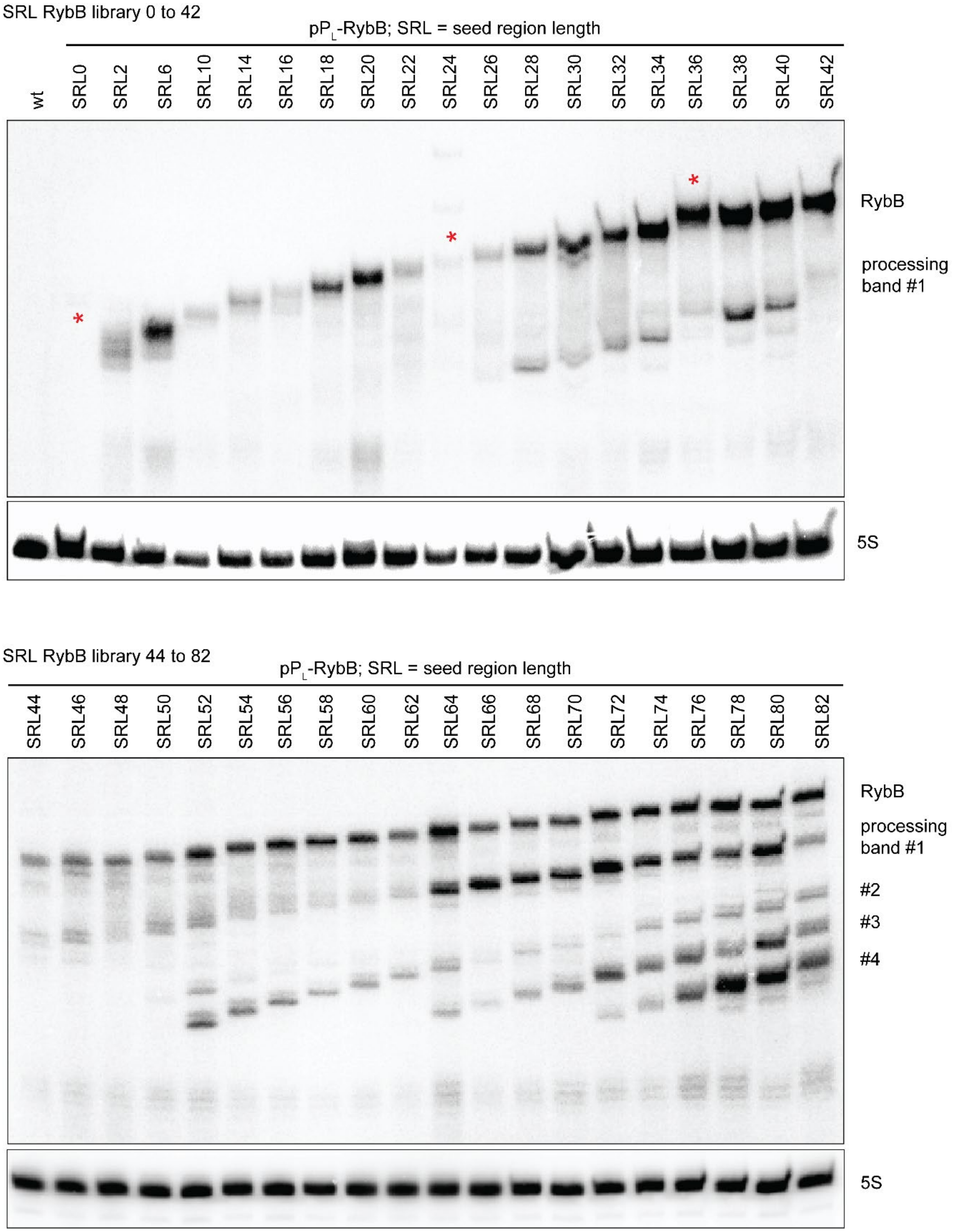

Figure S2 | Northern blot analysis of the RybB SRL-library, related to Figure 2-4. Northern blot analysis reveals a distinct processing pattern with the first band appearing with SRL26, the second with SRL52, the third with SRL64 and the fourth with SRL72. Intensities of synthetic sRNAs and processing patterns do not correlate with functionality (*cf*. Figure 2B). Red asterisk indicate sRNAs which are not expressed (SRL0), are potentially a mixed population (SRL24) or are the wrong size (SRL36). 5S rRNA serves as a loading control.

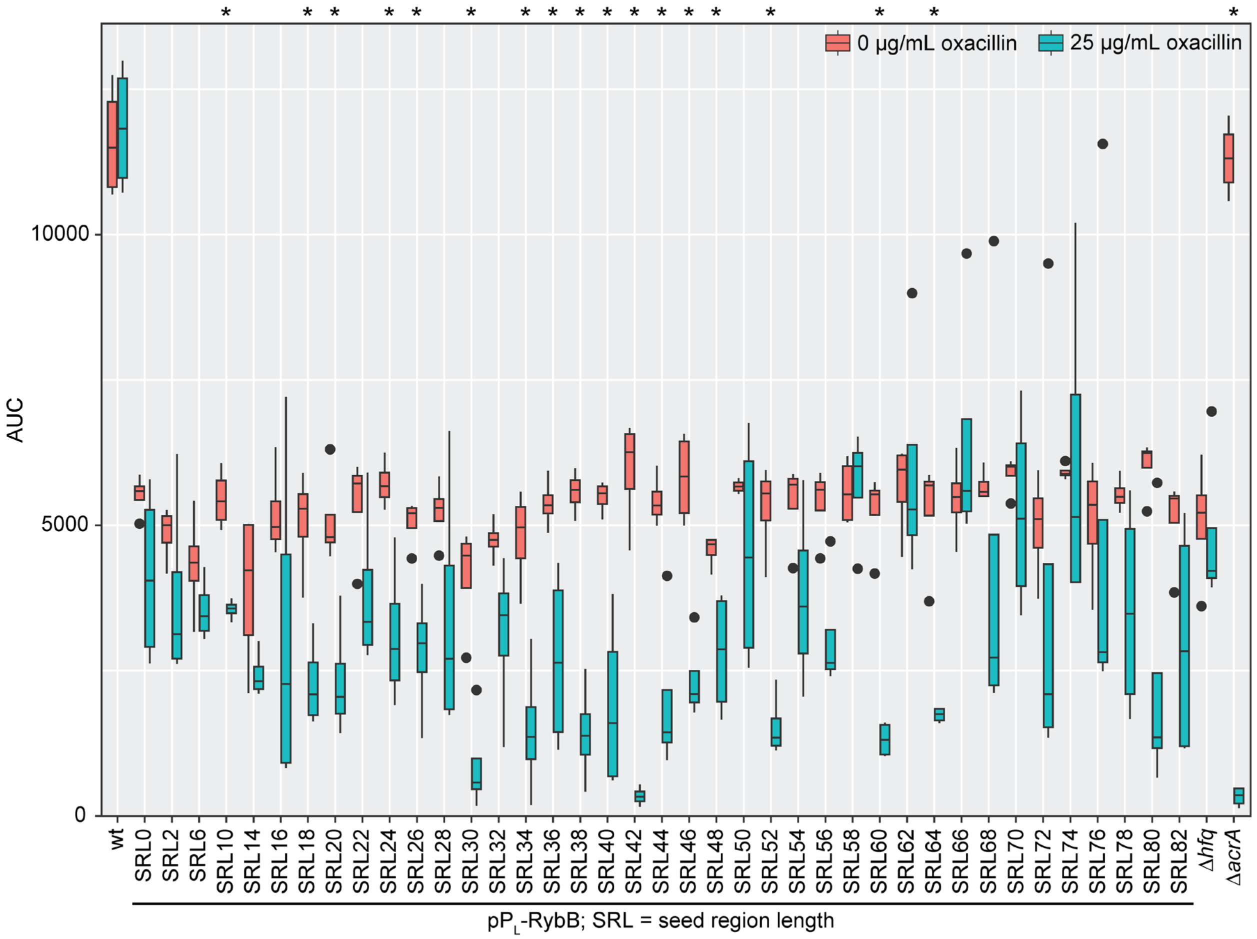

Figure S3 | Determination of synthetic RybB sRNAs functionality in Δ*hfq*, related to Figure 3. Liquid growth analysis of the SRL-library indicates no clear regulation for the synthetic sRNAs by accessing the area under the curve (AUC) in the absence and presence of oxacillin (25 µg/mL). The Δ*hfq* strain has a reduced viability, the AUC is decreased by approximately 2-fold even in the absence of oxacillin when compared to the wild type (wt). An increased SRL of the sRNAs does not show an effect. Wild type (wt), Δ*hfq* and Δ*acrA* with an empty plasmid serve as positive and negative controls. Oxacillin susceptibility assay was performed in quadruplicate. Wilcoxon-Mann-Whitney-Test was applied for statistical testing against the Δ*hfq* reference strain; $p<0.05$ (non-parametric, unpaired).

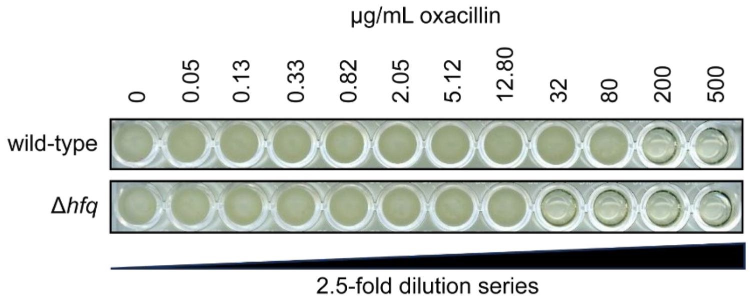

Figure S4 | MIC determination for the *hfq* deletion strain, related to Figure 3 and Figure 7. Stationary-phase cultures were diluted 1,000-fold and loaded into 96-well plates. Oxacillin was present at the indicated concentrations (2.5-fold dilution series starting at 500 µg/mL). A well without oxacillin was used as growth control. The 96-well plates were incubated at 37°C under continuous shaking for 24 h. A representative experiment is shown.

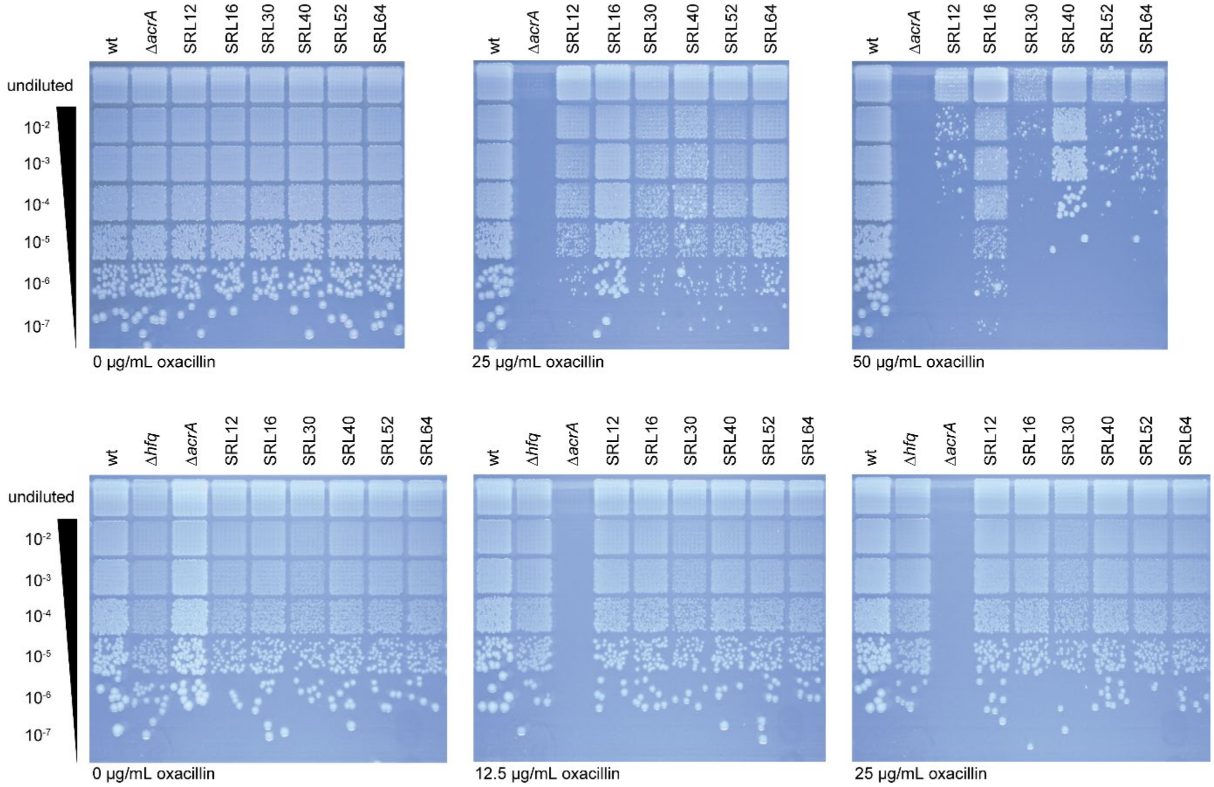

Figure S5 | Solid growth oxacillin susceptibility assay for selected candidates in wild type and Δ*hfq*, *r*elated to Figure 3. Upper panels show sRNA functionality in the wild type (wt). The lower panels show oxacillin susceptibility for Δ*hfq* expressing the indicated synthetic RybB sRNAs. No regulation of the *acrA* target can be observed in Δ*hfq*, which is consistent with the liquid media susceptibility assay (Figure 3A). Based on the reduced viability of Δ*hfq* in contrast to the wild type, the concentration of oxacillin for Δ*hfq* assays was reduced by half (Figure S4). A representative replicate is shown.

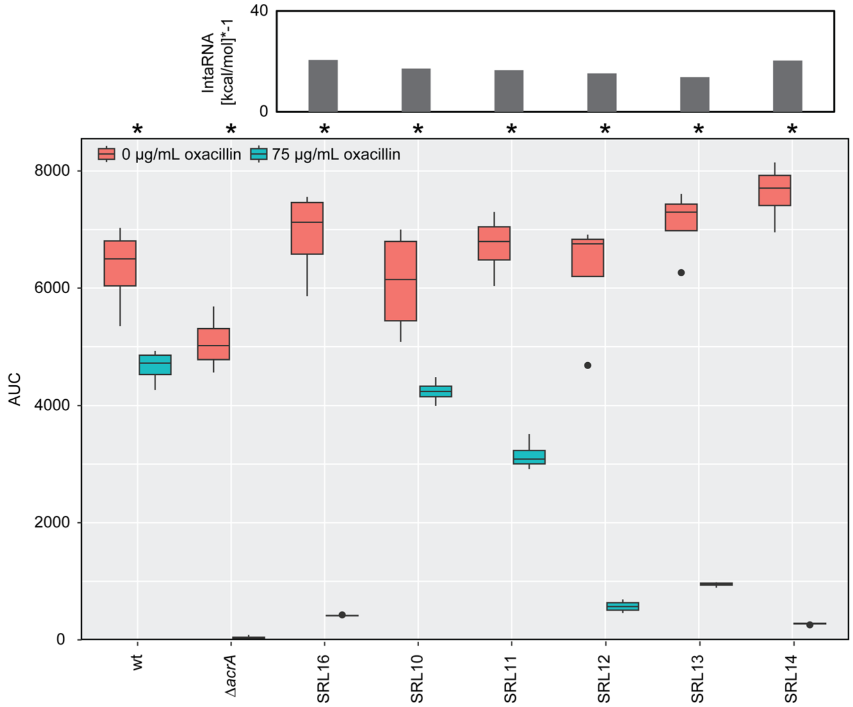

Figure S6 | Liquid oxacillin susceptibility assay for minimal seed region length of synthetic SgrS sRNAs, related to Figure 5. Oxacillin susceptibility assay was performed in quadruplicate. Wilcoxon-Mann-Whitney-Test was used for statistical testing of the difference between growth with and without oxacillin; p<0.05 (non-parametric, unpaired).

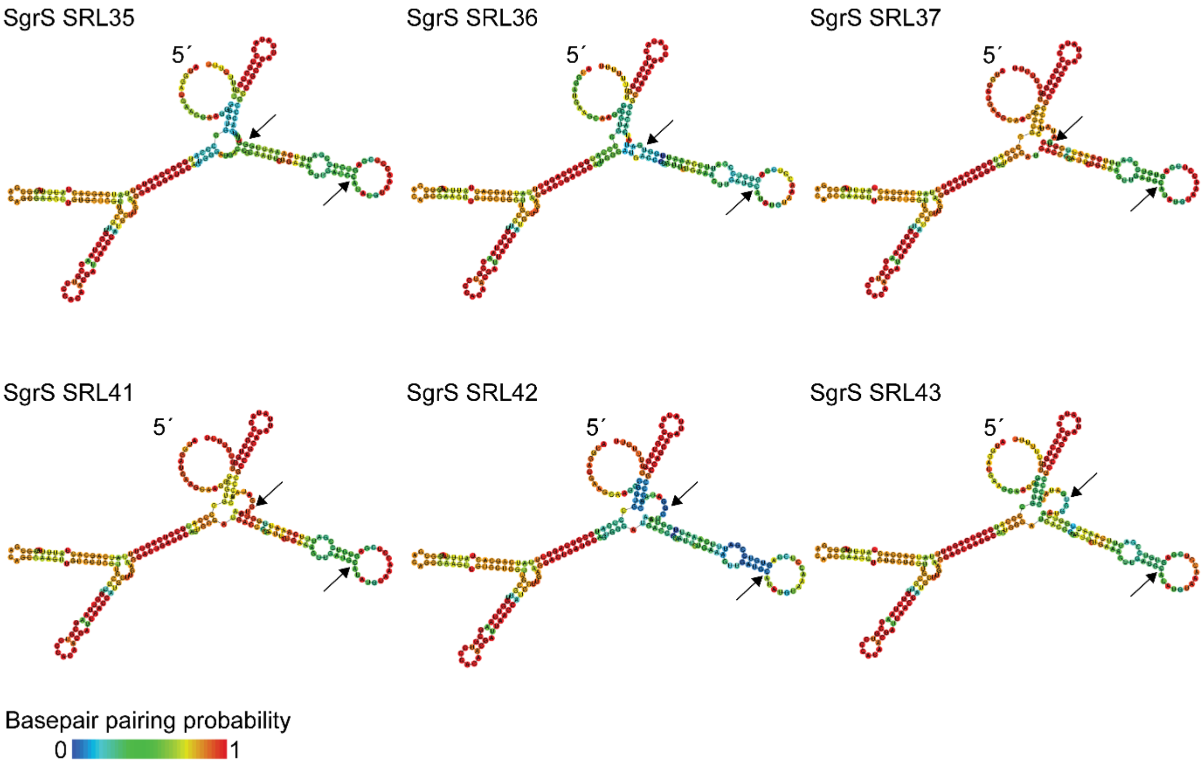

Figure S7 | Structure predictions of selected synthetic SgrS sRNAs, related to Figure 6. The seed regions of SgrS SRL35-37 and SRL41-43 are in a flexible stem loop structure with a comparably low base-pairing probability (seed regions are indicated by black arrows). The stem-loop structures of SgrS SRL37 and 41 have a higher base-pairing probability than the remaining SgrS sRNAs. Structures were generated using RNAfold [S1].

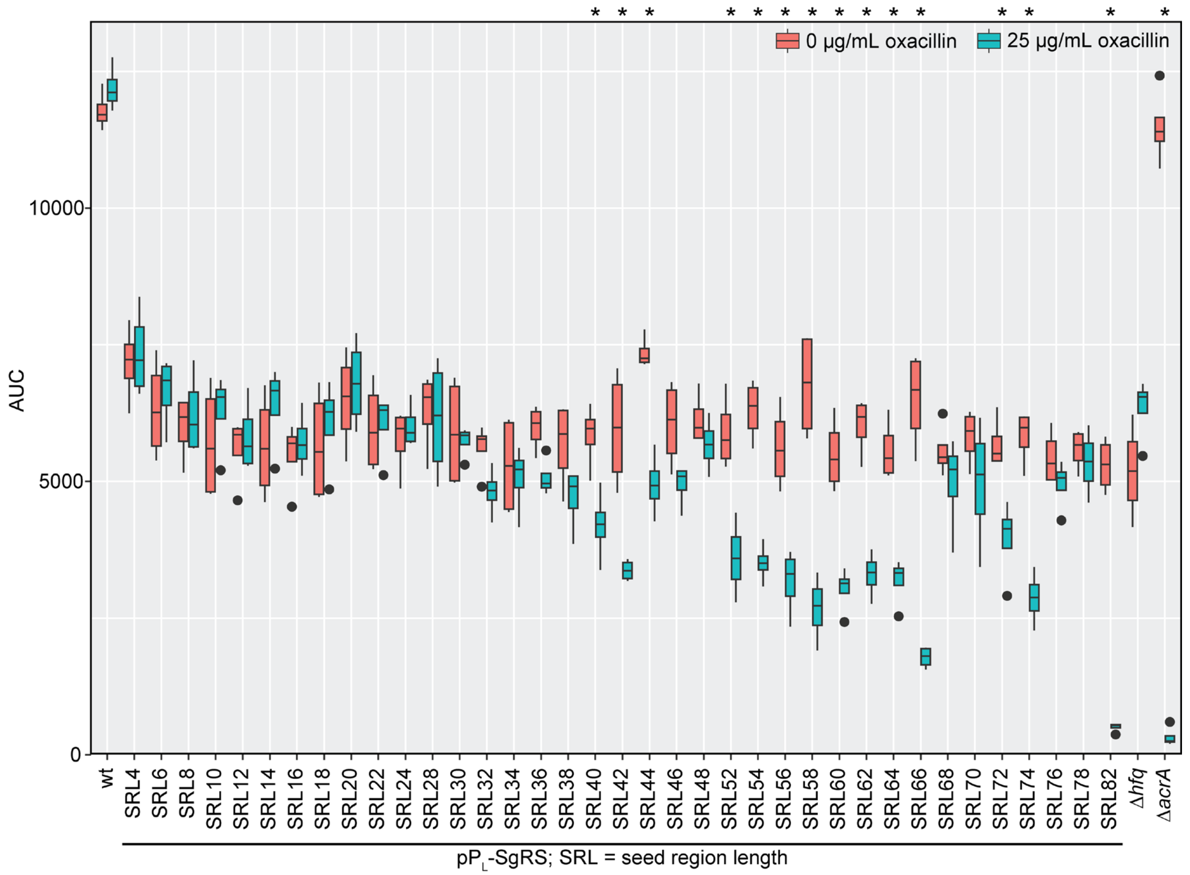

Figure S8 | Determination of synthetic SgrS sRNAs functionality in Δ*hfq*, related to Figure 7. Liquid growth analysis of the SgrS SRL-library indicates no strong regulation for the synthetic sRNAs by accessing the area under the curve (AUC) in the absence and presence of oxacillin (25 µg/mL). Increasing the SRL shows a mild correlation with reduced AUC in the presence of oxacillin. Notably, the best performing SgrS sRNAs (SRL36 and SRL42) are not the best regulating sRNAs in the absence of Hfq. Wild type (wt), Δ*hfq* and Δ*acrA* with an empty plasmid serve as respective positive and negative controls. Oxacillin susceptibility assay was performed in quadruplicate. Wilcoxon-Mann-Whitney-Test was used for statistical testing of the difference between growth with and without oxacillin; $p<0.05$ (non-parametric, unpaired).

Table S1 | Strains used in this study, related to STAR Methods.

Name	Relevant features	Reference
E. coli K-12 MG1655	K-12 F⁻ λ⁻	[S2]
E. coli DB3.1	F⁻ *gyrA462 endA1 glnV44* Δ(*sr1-recA*) *mcrB mrr hsdS20*(r_B^-, m_B^-) *ara14 galK2 lacY1 proA2 rpsL20*(Str^R) *xyl5* Δ*leu mtl1*	Invitrogen
E. coli Top10	F⁻ *mcrA* Δ(*mrr-hsdRMS-mcrBC*) φ80*lacZ*ΔM15 Δ*lacX74 nupG recA1 araD139* Δ(*ara-leu*)7697 *galE15 galK16 rpsL*(Str^R) *endA1* λ⁻	Invitrogen
Δ*acrA*	MG1655 Δ*acrA*::*cat*, Cm^R	[S3]
Δ*hfq*	MG1655 Δ*hfq*::*cat*, Cm^R	this study
Δ*rybB*	MG1655 Δ*rybB*::*cat*, Cm^R	[S3]
acrA-9′-syfp2	MG1655 *acrA-9′-syfp2-cat*, translational fusion of first 9 *acrA* codons to *syfp2*, Cm^R	[S3]
Δ*hfq::FRT acrA-9'-syfp2-cat*	MG1655 Δ*hfq::FRT acrA-9'-syfp2-cat*	this study
E. coli BL321	$RNaseIII^-$ (*rnc*⁻) *nadB*⁺ *purI*⁺	[S4]
E. coli BL322	$RNaseIII^+$ (*rnc*⁺) *nadB*⁺ *purI*⁺	[S4]
E. coli N3431	*lacZ43 relA1 spoT1 thi-1 rne-3071*	[S5]
E. coli N3433	*lacZ43 relA1 spoT1 thi-1*	[S5]
N3431 Δ*rybB*	*lacZ43 relA1 spoT1 thi-1 rne-3071* Δ*rybB*::*cat*, Cm^R	this study
N3433 Δ*rybB*	*lacZ43 relA1 spoT1 thi-1* Δ*rybB*::*cat*, Cm^R	this study

Table S2 | Oligonucleotide sequences used and created in this study, related to STAR Methods.

ID	Sequence 5´ -> 3´	Purpose
SLo0765	TGAAGAGCAGGCACGAACCC	Forward primer to amplify level 0 plasmid for blunt cloning, based on e.g., pSL099; expected size 2.1 kb
SLo0766	AGAAGAGCGAGCACAGAGTGC	Reverse primer to amplify level 0 plasmid for blunt cloning, based on e.g., pSL099; expected size 2.1 kb
SLo1503	TTCTTGTGAGCGGATAACAATTGACATTGTGAGCGGATAACAAGATACTGAGCACCCATG	Forward primer for P_L promoter generation in combination with SLo1504 for annealing into level 0
SLo1504	CATGGGTGCTCAGTATCTTGTTATCCGCTCACAATGTCAATTGTTATCCGCTCACAAGAA	Reverse primer for P_L promoter generation in combination with SLo1503 for annealing into level 0
SLo1505	GGAAAGCTAGCTCTTCCATG	Forward primer to amplify oligonucleotide library with SapI site with primer SLo1506
SLo1506	CACAGCTTAGCTCTTCGATC	Reverse primer to amplify oligonucleotide library with SapI site with primer SLo1505
SLo1510	GATGTCCCCATTTTGTGGAG	Forward primer to amplify *rybB* scaffold and terminator with primer SLo1512
SLo1511	ATGGATGTCCCCATTTTGTGGAG	Forward primer to amplify *rybB* scaffold and terminator with primer SLo1512
SLo1512	CCGGGATGACGCTGCATTTTGTC	Reverse primer to amplify *rybB* scaffold and terminator with primer SLo1510 or SLo1511
SLo1521	ATGGATGAAGCAAGGGGGTGCCC	Forward primer to amplify wt *sgrS* and terminator with primer SLo1522
SLo1522	CCGTCATAAAAGCGACCAGCATAAATGC	Reverse primer to amplify *sgrS* scaffold and terminator with primer SLo1522, SLo1523 or SLo1524
SLo1523	GATATCACCCGCCAGCAGATTATACC	Forward primer to amplify *sgrS* scaffold and terminator with primer SLo1520
SLo1524	TCAACTTTCAGAATTGCGGTC	Overlap extension PCR primer with SLo1521 to generate subsequently with amplicon SLo1522/1525 for *sgrS*
SLo1525	GACCGCAATTCTGAAAGTTGAATCACCCGCCAGCAGATTATACC	Overlap extension PCR primer with SLo1522 to generate subsequently with amplicon SLo1521/1524 for *sgrS*
SLo1526	TTCTTGTGAGCGGATAACAATTGACATTGTGAGCGGATAACAAGATACTGAGCACCCATGGATGAAGCAAGGGGGTGCCC	Forward primer to generate promoter 5´ *sgrS* scaffold with SLo1527
SLo1527	CATCAACTTTCAGAATTGCGGTC	Reverse primer to generate promoter 5´-*sgrS* scaffold with SLo1526
SLo1577	CCCAGTCACGACGTTGTAAAACGCGTCAATTGTCTGATTCGTTACCA	Forward sRNA nanopore sequencing primer for barcoding; single TU constructs based on pSL137 – M13 barcoding compatible
SLo1578	AGCGGATAACAATTTCACACAGGCTTCTCTCATCCGCCAAAACA	Reverse sRNA nanopore sequencing primer for barcoding; single TU constructs based on pSL137 – M13 barcoding compatible
SLo5132	ATGCATATGTAAAC	Forward oligonucleotide for seed region with 11 nt
SLo5133	ATCGTTTACATATG	Reverse oligonucleotide for seed region with 11 nt

ID	Sequence 5´ -> 3´	Purpose
SLo5134	ATGCATATGTAAACC	Forward oligonucleotide for seed region with 12 nt
SLo5135	ATCGGTTTACATATG	Reverse oligonucleotide for seed region with 12 nt
SLo5136	ATGCATATGTAAACCT	Forward oligonucleotide for seed region with 13 nt
SLo5137	ATCAGGTTTACATATG	Reverse oligonucleotide for seed region with 13 nt
SLo5138	ATGCATATGTAAACCTCGAGTGTCCGATTTCAAA TTGG	Forward oligonucleotide for seed region with 35 nt
SLo5139	ATCCCAATTTGAAATCGGACACTCGAGGTTTACA TATG	Reverse oligonucleotide for seed region with 35 nt
SLo5140	ATGCATATGTAAACCTCGAGTGTCCGATTTCAAA TTGGTC	Forward oligonucleotide for seed region with 37 nt
SLo5141	ATCGACCAATTTGAAATCGGACACTCGAGGTTTA CATATG	Reverse oligonucleotide for seed region with 37 nt
SLo5142	ATGCATATGTAAACCTCGAGTGTCCGATTTCAAA TTGGTCAATG	Forward oligonucleotide for seed region with 41 nt
SLo5143	ATCCATTGACCAATTTGAAATCGGACACTCGAGG TTTACATATG	Reverse oligonucleotide for seed region with 41 nt
SLo5144	ATGCATATGTAAACCTCGAGTGTCCGATTTCAAA TTGGTCAATGGT	Forward oligonucleotide for seed region with 43 nt
SLo5145	ATCACCATTGACCAATTTGAAATCGGACACTCGA GGTTTACATATG	Reverse oligonucleotide for seed region with 43 nt
acrAB-scr-1	GTATGTACCATAGCACGACG	Screening of *acrA* and *acrAB* manipulations
hfq-KO-1	AAGGTTCAAAGTACAAATAAGCATATAAGGAAAA GAGAGATGTAGGCTGGAGCTGCTTC	Deletion of *hfq* by Lambda Red recombineering
hfq-KO-2	AGGATCGCTGGCTCCCCGTGTAAAAAAACAGCCC GAAACCCTCATATGAATATCCTCCTTAGTTCC	Deletion of *hfq* by Lambda Red recombineering
hfq-scr-1	GTTTATCGAGGTGTTTGCGC	Screening of *hfq* deletion
hfq-scr-2	ATCACCTGCAATGCTTCGAC	Screening of *hfq* deletion
sYFP2_out	CGCGTCTTGTAGTTACCG	Screening of sYFP2 reporter gene
5S probe-2	CCTGGCAGTTCCCTACTCTCGCATGAGGAG	End-labeling for detection of 5S rRNA
RybB-probe-2	GAAATGGCGGGGTTGATGGGCTCCACAAAATGGG GACATC	End-labeling for detection of RybB; binding after 16-nt seed region
SgrS-3’probe	AAAAAAAACCAGCAGGTATAATCTGCTGGCGGGT GAT	End-labeling for detection of SgrS (binds to 3' end)

Table S3 | Oligonucleotide sequences within the seed region oligo pool (SLop2), related to STAR Methods.

ID	Sequence 5´ -> 3´ *
SLop2.01	ggaaagctaGCTCTTCc**ATGGAT**cGAAGAGCtaagctgtg
SLop2.02	ggaaagctaGCTCTTCc**ATG**ca**GAT**cGAAGAGCtaagctgtg
SLop2.03	ggaaagctaGCTCTTCc**ATG**cata**GAT**cGAAGAGCtaagctgtg
SLop2.04	ggaaagctaGCTCTTCc**ATG**catatg**GAT**cGAAGAGCtaagctgtg
SLop2.05	ggaaagctaGCTCTTCc**ATG**catatgta**GAT**cGAAGAGCtaagctgtg
SLop2.06	ggaaagctaGCTCTTCc**ATG**catatgtaaa**GAT**cGAAGAGCtaagctgtg
SLop2.07	ggaaagctaGCTCTTCc**ATG**catatgtaaacc**GAT**cGAAGAGCtaagctgtg
SLop2.08	ggaaagctaGCTCTTCc**ATG**catatgtaaacctc**GAT**cGAAGAGCtaagctgtg
SLop2.09	ggaaagctaGCTCTTCc**ATG**catatgtaaacctcga**GAT**cGAAGAGCtaagctgtg
SLop2.10	ggaaagctaGCTCTTCc**ATG**catatgtaaacctcgagt**GAT**cGAAGAGCtaagctgtg
SLop2.11	ggaaagctaGCTCTTCc**ATG**catatgtaaacctcgagtgt**GAT**cGAAGAGCtaagctgtg
SLop2.12	ggaaagctaGCTCTTCc**ATG**catatgtaaacctcgagtgtcc**GAT**cGAAGAGCtaagctgtg
SLop2.13	ggaaagctaGCTCTTCc**ATG**catatgtaaacctcgagtgtccga**GAT**cGAAGAGCtaagctgtg
SLop2.14	ggaaagctaGCTCTTCc**ATG**catatgtaaacctcgagtgtccgatt**GAT**cGAAGAGCtaagctgtg
SLop2.15	ggaaagctaGCTCTTCc**ATG**catatgtaaacctcgagtgtccgatttc**GAT**cGAAGAGCtaagctgtg
SLop2.16	ggaaagctaGCTCTTCc**ATG**catatgtaaacctcgagtgtccgatttcaa**GAT**cGAAGAGCtaagctgtg
SLop2.17	ggaaagctaGCTCTTCc**ATG**catatgtaaacctcgagtgtccgatttcaaat**GAT**cGAAGAGCtaagctgtg
SLop2.18	ggaaagctaGCTCTTCc**ATG**catatgtaaacctcgagtgtccgatttcaaattg**GAT**cGAAGAGCtaagctgtg
SLop2.19	ggaaagctaGCTCTTCc**ATG**catatgtaaacctcgagtgtccgatttcaaattggt**GAT**cGAAGAGCtaagctgtg
SLop2.20	ggaaagctaGCTCTTCc**ATG**catatgtaaacctcgagtgtccgatttcaaattggtca**GAT**cGAAGAGCtaagctgtg
SLop2.21	ggaaagctaGCTCTTCc**ATG**catatgtaaacctcgagtgtccgatttcaaattggtcaat**GAT**cGAAGAGCtaagctgtg
SLop2.22	ggaaagctaGCTCTTCc**ATG**catatgtaaacctcgagtgtccgatttcaaattggtcaatgg**GAT**cGAAGAGCtaagctgtg
SLop2.23	ggaaagctaGCTCTTCc**ATG**catatgtaaacctcgagtgtccgatttcaaattggtcaatggtc**GAT**cGAAGAGCtaagctgtg
SLop2.24	ggaaagctaGCTCTTCc**ATG**catatgtaaacctcgagtgtccgatttcaaattggtcaatggtcaa**GAT**cGAAGAGCtaagctgtg
SLop2.25	ggaaagctaGCTCTTCc**ATG**catatgtaaacctcgagtgtccgatttcaaattggtcaatggtcaaaa**GAT**cGAAGAGCtaagctgtg
SLop2.26	ggaaagctaGCTCTTCc**ATG**catatgtaaacctcgagtgtccgatttcaaattggtcaatggtcaaaagt**GAT**cGAAGAGCtaagctgtg
SLop2.27	ggaaagctaGCTCTTCc**ATG**catatgtaaacctcgagtgtccgatttcaaattggtcaatggtcaaaagtta**GAT**cGAAGAGCtaagctgtg
SLop2.28	ggaaagctaGCTCTTCc**ATG**catatgtaaacctcgagtgtccgatttcaaattggtcaatggtcaaaagttaat**GAT**cGAAGAGCtaagctgtg
SLop2.29	ggaaagctaGCTCTTCc**ATG**catatgtaaacctcgagtgtccgatttcaaattggtcaatggtcaaaagttaataa**GAT**cGAAGAGCtaagctgtg

ID	Sequence 5´ -> 3´ *
SLop2.30	ggaaagctaGCTCTTCc**ATG**catatgtaaacctcgagtgtccgatttcaaattggtcaatggtcaaaagttaataaac**GAT**cGAAGAGCtaagctgtg
SLop2.31	ggaaagctaGCTCTTCc**ATG**catatgtaaacctcgagtgtccgatttcaaattggtcaatggtcaaaagttaataaaccc**GAT**cGAAGAGCtaagctgtg
SLop2.32	ggaaagctaGCTCTTCc**ATG**catatgtaaacctcgagtgtccgatttcaaattggtcaatggtcaaaagttaataaacccat**GAT**cGAAGAGCtaagctgtg
SLop2.33	ggaaagctaGCTCTTCc**ATG**catatgtaaacctcgagtgtccgatttcaaattggtcaatggtcaaaagttaataaacccattg**GAT**cGAAGAGCtaagctgtg
SLop2.34	ggaaagctaGCTCTTCc**ATG**catatgtaaacctcgagtgtccgatttcaaattggtcaatggtcaaaagttaataaacccattgct**GAT**cGAAGAGCtaagctgtg
SLop2.35	ggaaagctaGCTCTTCc**ATG**catatgtaaacctcgagtgtccgatttcaaattggtcaatggtcaaaagttaataaacccattgctgc**GAT**cGAAGAGCtaagctgtg
SLop2.36	ggaaagctaGCTCTTCc**ATG**catatgtaaacctcgagtgtccgatttcaaattggtcaatggtcaaaagttaataaacccattgctgcgt**GAT**cGAAGAGCtaagctgtg
SLop2.37	ggaaagctaGCTCTTCc**ATG**catatgtaaacctcgagtgtccgatttcaaattggtcaatggtcaaaagttaataaacccattgctgcgttt**GAT**cGAAGAGCtaagctgtg
SLop2.38	ggaaagctaGCTCTTCc**ATG**catatgtaaacctcgagtgtccgatttcaaattggtcaatggtcaaaagttaataaacccattgctgcgtttat**GAT**cGAAGAGCtaagctgtg
SLop2.39	ggaaagctaGCTCTTCc**ATG**catatgtaaacctcgagtgtccgatttcaaattggtcaatggtcaaaagttaataaacccattgctgcgtttatat**GAT**cGAAGAGCtaagctgtg
SLop2.40	ggaaagctaGCTCTTCc**ATG**catatgtaaacctcgagtgtccgatttcaaattggtcaatggtcaaaagttaataaacccattgctgcgtttatatta**GAT**cGAAGAGCtaagctgtg
SLop2.41	ggaaagctaGCTCTTCc**ATG**catatgtaaacctcgagtgtccgatttcaaattggtcaatggtcaaaagttaataaacccattgctgcgtttatattatc**GAT**cGAAGAGCtaagctgtg
SLop2.42	ggaaagctaGCTCTTCc**ATG**catatgtaaacctcgagtgtccgatttcaaattggtcaatggtcaaaagttaataaacccattgctgcgtttatattatcgt**GAT**cGAAGAGCtaagctgtg

* underlined nucleotides = SapI recognition site, **bold nucleotides** = restriction site

Table S4 | Plasmids used and created in this study, plasmid files except pSIM5 and 709-FLPe are available in GenBank format in Data S1, related to STAR Methods.

ID	Relevant features	Parental plasmid	Reference
pSIM5	λ red expression vector, pSC101 *ori*, $repA^{ts}$, Tet^R		[S6]
709-FLPe	FLPe expression plasmid, pSC101-ts *ori*, Amp^R		Gene Bridges GmbH
p-PL-RybB-s8	RybB s8, Kan^R		[S3]
pSLcol_05	RybB SRL library, Kan^R	pSL137	this study
pSLcol_08	SgrS SRL library, Kan^R	pSL137	this study
pSL009	Empty control vector, Kan^R	pBAD	[S3]
pSL099	Level 0 plasmid for subcloning of fragments to be released with SapI; $Spec^R$	pMA60 [S7]	[S8]
pSL123	RybB scaffold with downstream region level 0 part; $Spec^R$	pSL099	this study
pSL132	3´ *sgrS* with downstream region level 0 part; $Spec^R$ (with downstream duplicated sequence)	pSL099	this study
pSL133	P_L promoter + 5´ *sgrS* level 0 part; $Spec^R$	pSL099	this study
pSL135	P_L promoter level 0 part; $Spec^R$	pSL099	this study
pSL137	Derivative of pBAD with SapI recognition site for Golden Gate cloning, Kan^R	pBAD	[S3]
pSL571	RybB SRL11, Kan^R	pSL137	this study
pSL572	RybB SRL12, Kan^R	pSL137	this study
pSL573	RybB SRL13, Kan^R	pSL137	this study
pSL598	3´ *sgrS* with downstream region level 0 part; $Spec^R$ (Duplicated downstream sequence removed)	pSL099	this study
pSL723	SgrS SRL11, Kan^R	pSL137	this study
pSL724	SgrS SRL13, Kan^R	pSL137	this study
pSL725	SgrS SRL35, Kan^R	pSL137	this study
pSL726	SgrS SRL37, Kan^R	pSL137	this study
pSL727	SgrS SRL41, Kan^R	pSL137	this study
pSL728	SgrS SRL43, Kan^R	pSL137	this study

Supporting References

S1. Lorenz, R., Bernhart, S.H., Honer Zu Siederdissen, C., Tafer, H., Flamm, C., Stadler, P.F., and Hofacker, I.L. (2011). ViennaRNA package 2.0. Algorithms Mol Biol *6*, 26. 10.1186/1748-7188-6-26.

S2. Blattner, F.R., Plunkett, G., 3rd, Bloch, C.A., Perna, N.T., Burland, V., Riley, M., Collado-Vides, J., Glasner, J.D., Rode, C.K., Mayhew, G.F., et al. (1997). The complete genome sequence of *Escherichia coli* K-12. Science *277*, 1453-1462. 10.1126/science.277.5331.1453.

S3. Köbel, T.S., Melo Palhares, R., Fromm, C., Szymanski, W., Angelidou, G., Glatter, T., Georg, J., Berghoff, B.A., and Schindler, D. (2022). An easy-to-use plasmid toolset for efficient generation and benchmarking of synthetic small RNAs in bacteria. ACS Synth Biol *11*, 2989-3003. 10.1021/acssynbio.2c00164.

S4. Studier, F.W. (1975). Genetic mapping of a mutation that causes ribonucleases III deficiency in *Escherichia coli*. J Bacteriol *124*, 307-316. 10.1128/jb.124.1.307-316.1975.

S5. Goldblum, K., and Apririon, D. (1981). Inactivation of the ribonucleic acid-processing enzyme ribonuclease E blocks cell division. J Bacteriol *146*, 128-132. 10.1128/jb.146.1.128-132.1981.

S6. Datta, S., Costantino, N., and Court, D.L. (2006). A set of recombineering plasmids for gram-negative bacteria. Gene *379*, 109-115. 10.1016/j.gene.2006.04.018.

S7. Schindler, D., Milbredt, S., Sperlea, T., and Waldminghaus, T. (2016). Design and assembly of DNA sequence libraries for chromosomal insertion in bacteria based on a set of modified MoClo vectors. ACS Synth Biol *5*, 1362-1368. 10.1021/acssynbio.6b00089.

S8. Brück, M., Berghoff, B.A., and Schindler, D. (2024). *In silico* design, *in vitro* construction, and *in vivo* application of synthetic small regulatory RNAs in bacteria. Methods Mol Biol *2760*, 479-507. 10.1007/978-1-0716-3658-9_27.
